# Supplementary material for: Revaccination in Age-Risk Groups with Sputnik V Is Immunologically Effective and Depends on the Initial Neutralizing SARS-CoV-2 IgG Antibodies Level
Source: Vaccines (Basel). 2022 Dec 30;11(1):90. doi: 10.3390/vaccines11010090 (PMC9861797; doi:10.3390/vaccines11010090)
Supplement: Supplementary file 1 [file vaccines-11-00090-s001.zip › vaccines-2081592-supplementary.pdf]

Supplementary

Table S1. The increasing coefficient in the group of 500-2000 BAU/ml with a smaller division

Antibody level before revaccination (BAU/мл)	Antibody level, BAU/мл			p (Wilcoxon criterion)	Antibody level increasing, times	
	n	After RV1, Me[IQR]	After RV2, Me[IQR]		After RV1, Me[IQR]	After RV2 Me[IQR]
Without COVID-19 history						
500-1000	540	724 [465 – 1080]	772 [523 – 1280]	p_{12}=0.033* p_{13}<0.001* p_{23}=0.072	1.00 [0.67 – 1.50]	1.10 [0.73 – 1.74]
1000-1500	277	1190 [875 – 1630]	1220 [780 – 1830]	p_{12}=1.000 p_{13}=0.357 p_{23}=0.405	0.96 [0.70 – 1.36]	1.00 [0.63 – 1.54]
1500-2000	145	1520 [811 – 2230]	1470 [946 – 2500]	p_{12}=0.252 p_{13}=1.000 p_{23}=0.687	0.86 [0.46 – 1.25]	0.87 [0.56 – 1.45]
With COVID-19 history						
500-1000	91	789 [465 – 1220]	781 [485 – 1225]	p_{12}=0.075 p_{13}=0.177 p_{23}=0.993	1.15 [0.71 – 1.52]	1.01 [0.70 – 1.67]
1000-1500	64	1115 [779.5 – 1390]	1260 [775.5 – 1840]	p_{12}=0.555 p_{13}=1.000 p_{23}=0.657	0.93 [0.66 – 1.14]	0.99 [0.68 – 1.36]
1500-2000	46	1505 [943 – 2070]	1310 [934 – 1750]	p_{12}=1.000 p_{13}=0.030* p_{23}=1.000	0.85 [0.62 – 1.22]	0.79 [0.58 – 1.03]
